# A Review on Flexural Properties of Wood-Plastic Composites

**DOI:** 10.3390/polym14193942

**Published:** 2022-09-21

**Authors:** Bingyu Jian, Sarah Mohrmann, Haitao Li, Yuanjie Li, Mahmud Ashraf, Jun Zhou, Xiaoyan Zheng

**Affiliations:** 1 College of Civil Engineering, Nanjing Forestry University, Nanjing 210037, China; 2 Joint International Research Laboratory for Bio Composite Building Materials and Structures, Nanjing Forestry University, Nanjing 210037, China; 3 Jiangsu Qianyu WPC Technology Co., Ltd., Yixing 214200, China; 4 Geelong Waurn Ponds Campus, Deakin University, Geelong, VIC 3216, Australia

**Keywords:** wood–plastic polymer, flexural property, surface treatment, compatibility

## Abstract

Wood–plastic composite (WPC) is a kind of composite material that is made of plastic and wood fiber or wood powder. Because it is mothproof, is resistant to corrosion, and has plasticity, among other advantages, it has been researched and used increasingly in building materials. The flexural property of WPC is an important subject in evaluating its mechanical properties. In this paper, wood–plastic raw materials and processing technology are introduced; the internal and external factors of WPC which affect the flexural properties are analyzed; the different ways of enhancing the bending capacity, including the surface pretreatment, addition of different modifiers (compatibility agent and coupling agent) etc. are summarized; and the methods of operation and strengthening effect are analyzed. This work provides a reference for further research in related fields.

## 1. Introduction

WPC offers environmental protection, the appearance and rigidity of wood, and the toughness and processability of plastic. Its main advantages include realizing the utilization of waste straw, wood, and other waste; safety and environmental protection; low water absorption; resistance to deformation or cracking and pleasing natural appearance. WPC can be used for decorative purposes and as building materials in outdoor paving and wall panels. Composites are widely used in engineering because of their low cost, high strength-to-weight ratio, and ease of manufacture [1]. However, shortcomings such as poor thermal stability and compatibility with other construction materials may hinder WPC’s wider usage in construction. WPC has double modal anisotropy and nonlinear viscoelastic mechanical properties and they show different stress-strain responses under tension and compression [2]. The raw materials used in WPC are predominantly thermoplastic polymer, wood fiber, wood powder, plastic matrix such as polyethylene (PE), polyvinyl chloride (PVC), polypropylene (PP), polystyrene (PS), and acrylonitrile-butadiene-styrene terpolymer (ABS) [3,4]. The raw material and matrix are molded by extrusion or remodeling. In addition, flame retardants, compatibilizers, and coupling agents are needed to improve the properties of WPC. To reduce the carbon footprint of the built environment [5], waste wood and recycled plastics can be used as raw materials to produce environmentally friendly WPC, which can meet the performance requirements of construction [6,7,8]. Green transformation of the construction industry depends on the environmental friendliness of building materials [9].

The performance of composite materials depends on the type and content of wood filler, the choice of polymer matrix, and the compatible technology and process parameters [10]. Since lignocellulosic materials are hydrophilic and plastic substrates are hydrophobic, the mechanical properties of lignocellulosic materials are not fully utilized due to the poor bonding effect during processing. Therefore, the main principle of improving the mechanical properties of lignocellulosic materials is to enhance the interface bonding force between materials and substrates. The main methods to enhance the interfacial bonding strength are surface pretreatment of substrate and raw material, including physical and chemical methods [11,12,13,14,15,16,17,18,19,20,21,22,23], as well as adding modifiers, including compatibilizers, coupling agents, etc. Other methods include adding nanoscale inorganic minerals for composite modification. Inorganic minerals with a small particle size have significant influence on the mechanical properties of WPC, mainly because inorganic minerals with a small particle size fill the gap between WPC and wood powder particles, and thus greatly improve the compatibility and bending strength of WPC [24]. For example, adding an appropriate amount of nano-SiO_2_ can improve the mechanical properties of WPC such as the bending properties and tensile strength to a certain extent. CNTs-enhanced WPC can also improve the tensile, flexural, and creep resistance of composites. Nano boron nitride and nanocellulose can significantly improve the impact toughness and strength of composites [25].

**Figure 1:**
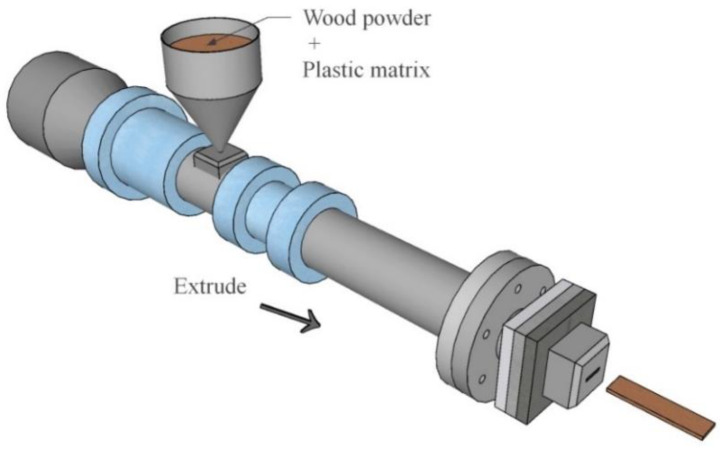
The extruder of WPC.

Therefore, the research on WPC mainly focused on the types of raw materials and processing techniques. Further research is needed on the influencing factors of bending resistance and the development of its application in engineering. This paper summarizes the types, proportions, and production methods of raw materials of WPC; compares the flexural properties of different types of WPC; and analyzes the effects of different strengthening methods on the flexural properties to provide reference for the enhancement and research on the flexural properties of WPC. A typical extruder used for WPC production is shown in Figure 1.

## 2. Influence of the Raw Materials

Biomass materials can replace traditional building materials for structural design and construction to a certain extent [26,27]. The mechanical properties of WPC are affected by the interfacial bonding force; significant research would be required to improve the interfacial bonding force to improve the mechanical properties of WPC. Some of the proposed solutions include surface pretreatment and addition of modifiers. As the main components of WPC are wood fiber and plastic matrix, physical or chemical surface pretreatment can enhance the interface binding force between the two. The physical methods include drying treatment, alkali treatment, and electric treatment, whilst the chemical methods include acylation of lignocellulosic, etherification, graft etc. Wood can be treated to broaden its application scope [28] in addition to use in building construction [29]. Wood can be used as a building material for multistorey buildings, where the structural configuration can be optimized for seismic design [30]. Arwinfar et al. [31] studied the influence of wood heat-treatment on the mechanical properties and morphological properties of WPC composites. The wood chips are first heated in a digester with saturated steam at 120 °C, 150 °C, or 180 °C for 30 to 120 min. The study showed that mechanical properties of beech wood were improved to varying extent with different heat-treatment times and temperatures. Experimental studies showed that the mechanical properties of WPC is best when heat treatment was done at150 °C for 30 min.

### 2.1. Influence of the Type of the Material

The properties (moisture content, type, density, etc.) and proportions of wood fiber and plastic matrix have an important influence on the bending strength of WPCs. Various researchers reported different techniques to enhance the bending strength by changing different manufacturing variables. 

Different types of WPCs made from wood fibers have different bending strengths. In recent years, researchers developed innovative sustainable materials that can boost the construction industry without causing damage to the environment [32]. Mu et al. [33] studied the preparation of HDPE matrix composites by using four biomass particles, namely poplar, pine, wheat straw, and bamboo as reinforcement fillers. Obtained results showed that poplar wood particles with high cellulose content had higher bending properties when combined with the HDPE matrix. Xu et al. [34] used poplar fiber and bamboo fiber as raw materials to prepare ultra-high filling PP-based WPC, and they made a comparative analysis of the effect of filling amount of wood fiber on mechanical properties at high- and low-temperature. Experimental results showed that the bending modulus increased first and then decreased at 90% when the filling amount was increased from 75% to 90%. Xu et al. [35] studied the influence of the mass ratio of Masson pine wood to Chinese fir wood on the bending properties of composite materials. Obtained results showed that the influence of Chinese fir on the flexural strength of the composite was greater than that of Masson pine. Compared with the Masson pine composite, the static flexural strength and modulus of the Chinese fir composite were increased by 47.58% and 25.00%, respectively. Liu [36] used the mechanical pulp of bagasse, natural chemical pulp of bagasse, and bleached chemical pulp as reinforcement and HDPE, respectively, to prepare pulp/HDPE WPC materials, and they explored the effects of different pulp-fiber raw materials on the properties of WPC. The mechanical properties of bagasse mechanical pulp/HDPE WPC showed the best mechanical properties, with a bending strength of 65.9 MPa. Calcium carbonate has been reported to offer good toughening effect on polymer and can be used as filler in WPC effectively [37].

### 2.2. Influence of Particle Size, Shape, and Amount of Wood Fiber

WPCs made of the same wood fibers with different particle sizes or shapes reportedly showed different bending strengths. Turku, Irina, et al. [38] compared the mechanical properties of composite materials prepared by three types of spruce wood powder (coarse powder, 20-mesh powder, and Arbocel C320 powder). The results showed that the composite made of 20-mesh wood powder had good bending properties. Chaudemanche et al. [39] reported that the bending strength and the bending modulus increased with the increase of wood powder particles. Leu et al. [40] used rPP plastic and wood-powder extrusion to prepare wood–plastic materials, and reported that finer wood powder (less than 125 μm) could improve the flexural strength of WPCs. 

Under bending load, when the rice husk content is less than 60 PHR, the bending modulus and ultimate strength showed a linearly proportional relationship but when the rice husk content was higher than 60 PHR, the modulus and strength was decreased due to the formation of rice husk aggregates [41]. Wang et al. [42] prepared high-performance WPC by reinforcing HDPE with waste-wood particles in needle-like, flak-like, strip, and powdery shapes and tested the mechanical properties. The experimental results showed that the wood needle with a large aspect ratio is most suitable for producing high-performance wood–plastic material. Schirp et al. [43] used beech wood and HDPE to make WPC with synthetic fiber (60% by weight) in an internal kneading mixer; it was found that, with the increase of fiber length, the water absorption rate of the WPC decreased, and the flexural strength and elastic modulus increased. Delviawan et al. [44] studied the effects of particle size distribution and temperature on the mechanical properties of WPC, and it was found that the best mechanical properties are obtained under 30 min wet grinding and freeze-drying condition. Arnandha et al. [45] found that the bending strength of WPC made from Sengong sawdust was approximately 40.49 MPa. Mertens et al. [46] studied the influence of fiber loading and fiber geometry on the mechanical properties of a wood–PP composite. When the fiber content was 50 wt.%, the strength of the composite reached the peak and the bending strength reached 76.4 MPa. 

Therefore, wood fibers with a large aspect ratio are suitable for making high-performance WPC. The relationship between particle size and flexural properties is related to the type of materials, and most of them are 60–80 mesh particles. 

**Figure 2:**
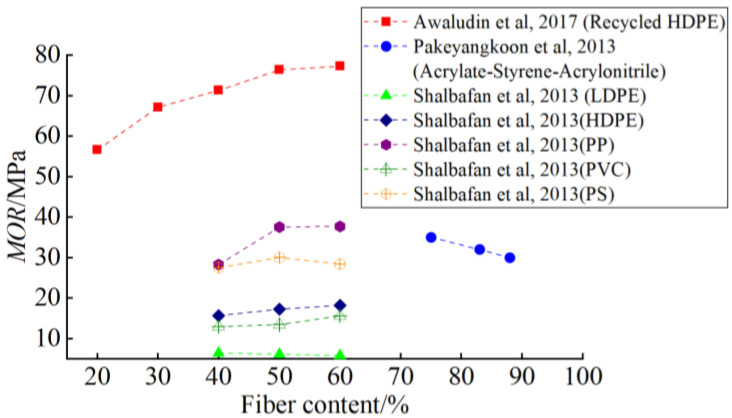
Wood fiber content and bending strength [45,48,49].

**Figure 3:**
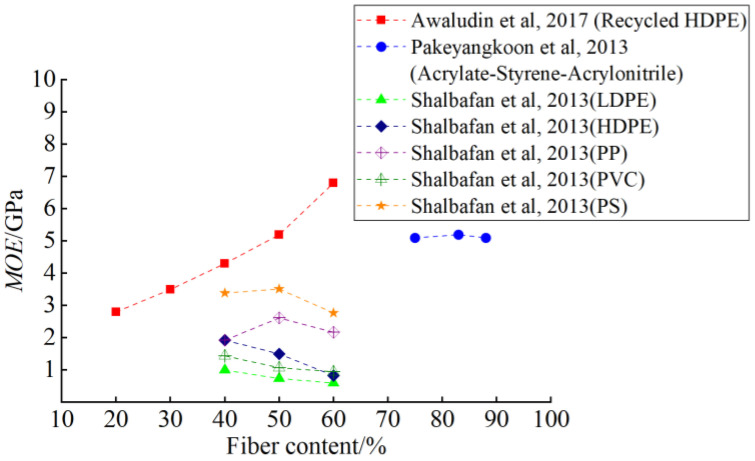
Wood fiber content and elastic properties of WPC [45,48,49].

The WPC bending strength is affected by the addition of wood fiber. Generally, within a reasonable proportion range, the bending strength increases with the addition of wood fiber up to a certain limit. One of the key research questions in studying the mechanical properties of WPC is to obtain the optimal ratio by testing different content. Barbos et al. [47] used HDPE as the matrix and added 60%, 65%, and 70% wood powder to make WPC. It was found that the bending strength decreased with the increase of wood-powder content, and the bending modulus reached its maximum at 65%. Pakeyangkoon et al. [48] studied the mechanical properties of WPC prepared from acrylic styrene acrylonitrile (ASA) and bagasse. WPCs with excellent mechanical properties were prepared from 10~50 PHR bagasse. It was found that the bending strength and bending modulus of WPC increased with the increase of bagasse content. Shalbafan et al. [49] used lightweight foam-core particleboard as a raw material to study the influence of different fiber content and plastic matrices on the flexural performance of WPC. Figure 2 and Figure 3 show the relationship between wood fiber content and flexural properties reported in previous studies [45,48,49]. It is worth noting that *MOR* means the modulus of rupture, and *MOE* means the modulus of elastic. By comparison, it is found that Awaludin et al [45] obtained the best flexural properties using HDPE in comparison to different plastic substrates used by Shalbafan et al [49].

Significant differences have been reported in the mechanical properties of WPC composites with different proportions. Higher the content of wood fiber increases the hardness of WPC, whilst increasing the proportion of plastic matrix increases the toughness of the composite material. Use of different type of wood fibers, in some studies, has been reported to produce higher mechanical properties when compared to a single fiber type. Ayrilmis et al. [50] studied the preparation of brown rot fungi from Scottish pine wood with different proportions (30%, 40%, or 50%) and PP coupling agent (maleic anhydride grafted PP, 3 wt.%). The results were compared with the properties of WPC made from solid wood. The flexural properties of decayed-wood powder were lower than those of normal wood powder, but the flexural properties of wood powder were better when the dosage of wood powder was 40% and 50%. Hosseinihashemi et al. [51] evaluated the individual and interaction effects of poplar endothelial powder (IBF), bark powder (OBF), and wood meal (WF), and the effect of WF content on the mechanical properties of WPCs. The results showed that WF supplementation significantly increased the bending strength, bending modulus, and tensile strength (*p* < 0.05) of WPC. Compared with WF alone and IBF/OBF alone, the IBF/WF composite showed a higher tensile modulus. With the same content, processing methods, and the plastics substrate as composites with cypress wood, Chinese fir showed good mechanical performance in WPCs. 

### 2.3. Influence of Plastic Matrix

The properties of plastic matrix and the amount of compatibilizer have been reported to show considerable influence on the bending strength of WPCs. Researchers have studied different types of plastic matrices to select the plastic matrix that meets the performance requirements of WPC. 

Environmental conditions have an important influence on the mechanical properties of plastic matrix. Ratanawilai et al. [3] studied the influence of plastic matrix on the flexural properties of WPC by using HDPE, LDPE, PP, PVC, and PS to prepare composites. It was reported that WPCs made of PS and PP had higher bending properties, while LDPE, HDPE, and PVC had lower bending properties. In particular, the mechanical properties of WPC made by using LDPE showed inferior mechanical properties. Hartmann et al. [52] used a formaldehyde-free binder system to prepare a wood-chip–plastic composite material (WCPC) with high mechanical properties, which can be an alternative to the common oriented particle board (OSB). WCPC is load and application optimized, non-toxic, zero waste, and cost-effective.

### 2.4. Influence of the Environmental Parameters

WPCs have different types of applications that require protection against varying environmental conditions including humidity, temperature, moisture content etc. Yang et al. [53] conducted freezing and heat treatment of WPC at different temperatures to study its influence on its size and mechanical properties. At a higher temperature, the WPC initially expanded rapidly and then contracted slowly until it reached equilibrium. At 52 °C and 50% relative humidity for 16 days, the bending strength was reported to increase by 8%. Jiang et al. [54] studied the influence of compression pressure and mold temperature on the bending strength, density, water absorption, and size stability. The results show that the bending strength and density increase with the increase of compression pressure.

Previous research showed that superior mechanical properties can be obtained when the diameter of wood fiber is between 0.21 and 0.29 mm (60–80 mesh) and the aspect ratio is between 5 and 8. The mechanical properties of pine-fiber-reinforced composites were better than those of poplar-fiber-reinforced composites [55]. Silane coupling agent KH590 could effectively improve the surface free energy of silicate-modified poplar [56]. Chen et al. [57] investigated the influence of the wood–plastic ratio on the mechanical properties of wood powder/high-density polyethylene (WF/HDPE) composites and prepared the composites by treating wood fiber with a silane coupling agent, adding MaH-G-HDPE compatibilizer to the polymer; the coupling agent and the compatibilizer were added simultaneously. Chen et al. [57] investigated the effects of three treatments on the mechanical properties of WF/HDPE composites. Experimental results showed that addition silane coupling agent A-171 or capacifying agent MaH-G-HDPE alone could improve the mechanical properties of WPC, but when those agents were used simultaneously the mechanical properties were further enhanced. Yu et al. [58] used wheat straw powder (WSP) and linear low-density polyethylene (LLDPE), HDPE, ABS, PS, and PP as raw materials to prepare WPC by using the extrusion and injection molding process. The mechanical properties of different WPCs were studied and the experimental results showed that the static flexural strength and static flexural modulus of HDPE- and PP-based WPCs were the best, followed by ABS- and PS-based WPC and LLDPE-based WPC. 

Different types of fibers have been reported to show different effects on the flexural property of WPC. Different wood has different adaptability to humidity, temperature, and weathering, and hence the selection of wood fiber species should be done considering the environment, climate, and performance requirements. The content and particle size of the same type of wood fiber generally have a peak of bending strength; that is, within this range, the bending strength increases with the increase of fiber content and the decrease of particle size. WPCs made with needle fibers with different fiber shapes and a large aspect ratio generally have a higher bending performance. The bending strength of WPCs made of different types of plastic matrix showed considerable variations. The types and proportions of raw materials reported in previous research with the corresponding bending properties are shown in Table 1. It is worth noting that that different types of of raw materials have different optimal WPC ratios but typically, use of 50–60% wood fiber content produced good bending resistance. The wood fiber content should be considered according to the type and nature of raw materials and the environment in which they are used. WPCs made of PS and PP offer superior resistance in bending, while those made of PE and PVC give relatively inferior performance in bending.

## 3. Surface Pretreatment

Biomass pretreatment is a necessary way to break the three-dimensional network structure of the plant cell wall and break the anti-degradation barrier of lignocellulose [68]. Surface pretreatment can be divided into physical and chemical methods. Physical methods include drying, alkali treatment, discharge treatment, and other ways to enhance the interface adhesion between lignocellulosic and plastic matrix. Hietala et al. [11] studied the influence of chemical pretreatment and water content on the mechanical properties of WPCs. It was found that the mechanical properties of WPC could be improved by extrusion of pretreated and dried sawdust. In practice, most of the polymers used in WPC show hydrophilic properties. Therefore, most researchers investigated the micro/nanostructures on the wood surface prior to using low-surface-energy materials for hydrophobic modification [67].

### 3.1. Physical Methods

Hydrophobic modification can improve the mechanical properties of WPC. Zhang et al. [12] carried out hydrophobic modification of wood flour, and it was found that the mechanical properties of WPC were significantly improved after hydrophobic modification. When compared with WPCs without hydrophobic modification, the bending strength increased by 17.3%, 26.3%, and 27.5%, and the bending modulus increased by 24.4%, 24.4%, and 26.0%, respectively. Hot-water extraction can reduce the quality and improve the mechanical properties of WPC. Pelaez-samaniego et al. [13] modified unpeeled Ponderosa pine slices by using hot-water extraction. After soaking for 2520 h, the bending test showed that the WPC performance was improved. Grubbstrom et al. [14] studied silane crosslinking of reclaimed LDPE wood composites and its effect on the properties of the composites. The results showed that the flexural strength of the uncrosslinked specimens was increased. Acosta et al. [15] used the vacuum-compression method and 1.5 wt.% benzoyl peroxide as an initiator to impregnate wood with vinyl acetate monomer, and the bending strength of the treated WPC was superior to that of the original. 

The mechanical properties of the raw sawdust composite showed similar mechanical as those produced using the dry sawdust composite although the former had higher aspect ratio [16]. Lou et al. [17] treated coconut bran with alkali to remove pectin, hemicellulose, fat, and impurities. In their study, wood–plastic composite was made of coconut shell fiber and PP. Alkali treatment reduced the weight of the coconut shell; longer alkali treatment with higher concentration resulted in greater the loss of coconut shell fiber. 

**Figure 4:**
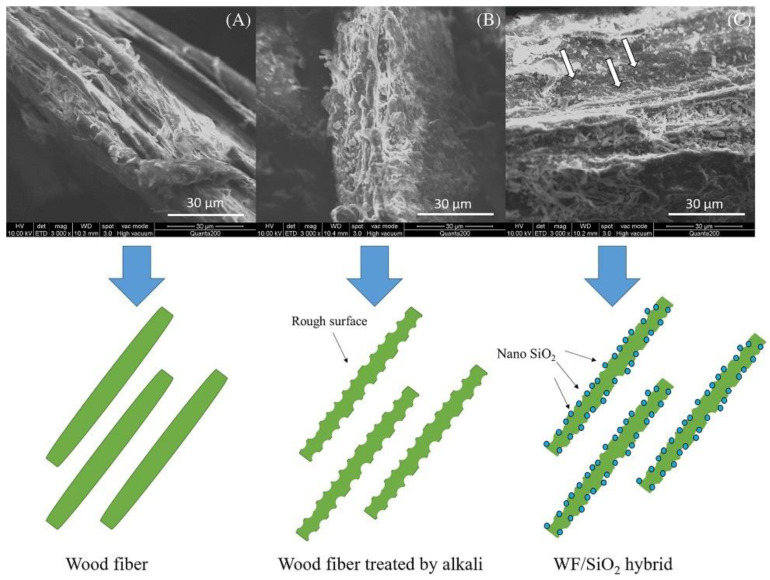
SEM morphology of (**A**) wood flour, (**B**) alkali-treated wood flour, and (**C**) WF/silica hybrid (adapted with permission from Ma. et al. [18]).

Alkali treatment can improve the mechanical properties of WPCs. Ma et al. [18] used alkali treatment and sol–gel method to modify wood powder to prepare wood powder/silica hybrid and use it as a reinforcing filler for PP-based WPC. The experimental results were compared with those of nano-silicon dioxide–PP-based WPC as shown in Figure 4.

Compared with industrial-silica-blended WPC, the WPC with mixed filler showed better mechanical properties. Chen et al. [19] reported that high-temperature hot-air treatment can promote the hydration of poplar fiber and improve the mechanical properties of WPC. The optimum mechanical properties were acheived at 220 °C.

### 3.2. Chemical Methods

Chemical methods such as acylation, etherification, or grafting of lignocellulosic fibers are often used to increase the interfacial bonding strength. Grafting copolymerization refers to the generation of free radicals by maleic anhydride, acrylonitrile, and other monomers under the action of initiators, and grafting copolymerization with plastic or lignocellulosic fibers to generate molecular chains with good compatibility with the matrix [20]. Furfuryl alcohol modification can improve the dimensional stability, mildew resistance, and hardness of modified wood [69].

Under dry conditions, the WPC composite containing the highest-molecular-weight MAPP has the highest bending modulus [59]. Li. [21] explored the influence of different contents of maleic anhydride grafted PE on the bending strength of glass fiber/wood plastic composite (GF/WPC), and the results showed that the maximum bending strength of the composite, after maleic anhydride grafted PE surface treatment, was 38.9 MPa, an increase of 21.6% when compared with that without the addition. Hong et al. [22] studied the mechanical properties and rheological properties of recycled polyethylene (rPE)/WPC, and the results showed that PE graft copolymer can significantly change the mechanical properties of rPE/WF. Gao et al. [60] prepared maleic anhydride grafted WPC, using PP, PE, PS, and other WPC composites as raw materials by reactive extrusion. The mechanical test results showed that the mechanical properties of the grafted plastic and waste plastic composites were significantly improved. Ramesh et al. [4] thoroughly mixed wood waste and phenolic formaldehyde in a rotary mixer to make samples of WPCs. The results showed that the chemically treated WPC has better properties than the untreated WPC. 

The grafting of wood fiber can improve the environmental adaptability of WPC. Li. [23] used hexyl trimethoxylsilane to conduct surface-grafting modification of plant fiber (PF) and prepared PE/modified-plant-fiber (MPF) WPC by PE and MPF. After 180 days, the bending strength and bending modulus decreased from 9.9% and 8.5% to 2.0% and 3.1%, respectively; the results indicated that the surface modification of PF can significantly improve the natural aging resistance of the WPC.

## 4. Addition of Modifier 

“Modifier”, typically, refers to the addition of compatibilizers. The connection between different kinds of materials in a building needs to consider the nature of the materials [70,71], and so does the complex. The compatibilizers used for WPC are mainly polymer resins with anhydride groups and carboxyl groups, such as maleic anhydride grafted PP, isocyanate, methylene succinic anhydride etc. The anhydride and carboxyl groups carried by the compatibilizer can react with the hydroxyl group on the surface of wood fiber to produce a chemical bond, while the non-polar or weak polar polymer chain is compatible with the resin and thus increases the compatibility between wood and plastic. Coupling agents (silane and titanic acid) can produce a strong interface bonding between the plastic and wood-fiber surface, reduce the water absorption of wood fiber, improve the compatibility and dispersion between wood fiber and plastic, and improve the mechanical properties of composite materials [20]. 

Lubricants and surfactants can also be added to enhance the interface adhesion between lignocellulosic and plastic substrates through chemical action. Perisic et al. [72] mixed polyethylene terephthalate (PET) fiber (raw fiber, waste fiber, and mixed fiber) into a PMMA–wood composite. Toluene-2, 4-diisocyanate (TDI), and (3-mercaptopropyl) trimethoxysilane (MPTMS) were used in the study as crosslinking agents to modify wood fibers. The mechanical-property test showed that the addition of PET fiber greatly improved the properties of the composites. 

Modifiers mainly include compatibilizers containing anhydride and carboxyl group; coupling agents contain silane and titanic acid, lubricants, surfactants, etc. The use of compatibilizers is an enhancement of the polarity of the plastic surface, as described in the surface pretreatment. The amount of modifier has a significant effect on the enhancement effect. Zhou et al. [73] used maleic anhydride polyethylene as a coupling agent to improve the interface adhesion between chopped carbon fiber and the plastic matrix. The mechanical properties of maleic anhydride polyethylene grafted composite material are 113~119% higher than that without maleic anhydride-grafted polyethylene, which is very close to the strength of structural wood. Bengtsson et al. [74] prepared silane crosslinked composites with different contents by using vinyl trimethoxysilane as the raw material. The composite was stored in sauna and room temperature to study the effect of humidity on crosslinking degree. Under the sauna condition, the crosslinking degree of the composites was the highest, and the mechanical properties of the crosslinking composites were better than those of the composites without silane. Hao et al. [75] found that, when compared with the incompatible composite with WF of 80 wt.%, the bending strength of the composite with MAPE as compatibilizer increased by 189%. Gao et al. [76] modified silane with a special chemical structure obtained from different types of poplar fibers. The effects of ICD-assisted silane modification on the wood properties of plastic composite materials (WPCs) were studied. The main results showed that the mechanical properties of WPC were significantly improved after ICD/silane co-modification with vinyl trimethoxysilane (A-171)-modified WPCs showing the best performance. The bending strength of 3% silane composite was increased by 10.22%. To achieve the best outcome, the compatibility between the modifier and the wood must be carefully considered in addition to the amount of modifier and the production process.

## 5. Other Methods

### 5.1. Modification by Adding Lignin

In addition to raw materials, other fiber powder, lignin, etc., can enhance the interface adhesion and thus enhance the bending strength. Li et al. [77] found that the mechanical strength of WPC prepared by adding lignin and coffee-shell powder was significantly improved, and the static flexural strength could be increased by 49.31%. The corrosion resistance of WPC was improved by adding lignin. When the content of alkali lignin was 15% and coffee-shell powder was 45%, the properties of WPC were the best. Sozen et al. [78] prepared composite materials by using PP as the polymer matrix, and wood powder and cellulose fiber as reinforcement fillers. The mechanical properties (bending strength and bending modulus) of the composites were studied, which showed that the flexural strength of the sawdust composites was higher than that of the cellulose composites, and the flexural modulus of the cellulose composites was higher than that of the wood-powder composites. 

Li et al. [79] prepared WPC by the hydroxylmethylation modification of alkali lignin and then modified alkali lignin, eucalyptus powder, and HDPE. The mechanical properties of WPC prepared by modified lignin were measured and analyzed. The results showed that the maximum flexural strength of WPCs prepared by modified lignin was increased by 37.68%.

Therefore, the addition of lignin or modified lignin can be regarded as a research direction to enhance the bending performance of WPCs. The types of lignin, modification methods, and the combination of lignin with raw materials and environment need to be further studied and developed.

### 5.2. Modification by Adding Nanomaterials

The addition of nanomaterials can fill the gaps between the wood fibers and increase the bending strength. The enhancement effect is related to the amount of additive, material type, and manufacturing process. An increase in density usually leads to an improvement in performance [80]. Rangavar et al. [81] studied the effect of nano-clay (NC) on the physical and mechanical properties of WPC. Raw PVC, recycled PVC, and mixed PVC (50/50% raw PVC/recycled PVC) were used as the WPC substrates. It was found that the highest performance was observed in the sample with 5% NC content. Kaymakci et al. [82] studied the effect of sepiolite clay nanofibers (SCNs) on the mechanical properties of wood–plastic nanocomposites. The results show that the bending properties of WPC composites increase with the increase of sepiolite nanofiber content. Wang [62] added trace (0.1–2%) micron aluminum powder into WPC as reinforcement, and it was found that when the content of aluminum powder was 0.1 wt.%, medium and high fire (539 W), 15 s, the mechanical properties were the best with an increase of 31.25% of bending strength reaching 16.8 MPa. Chen et al. [63] fused and molded the modified multi-walled carbon nanotubes with HDPE and wood powder, and tested the mechanical properties of the prepared composite material samples. The results showed that the bending strength and elastic modulus of the composite were increased by 5.8% and 13.7%, respectively, by a-171 surface-modified MWCNTs compared with untreated MWCNTs.

**Figure 5:**
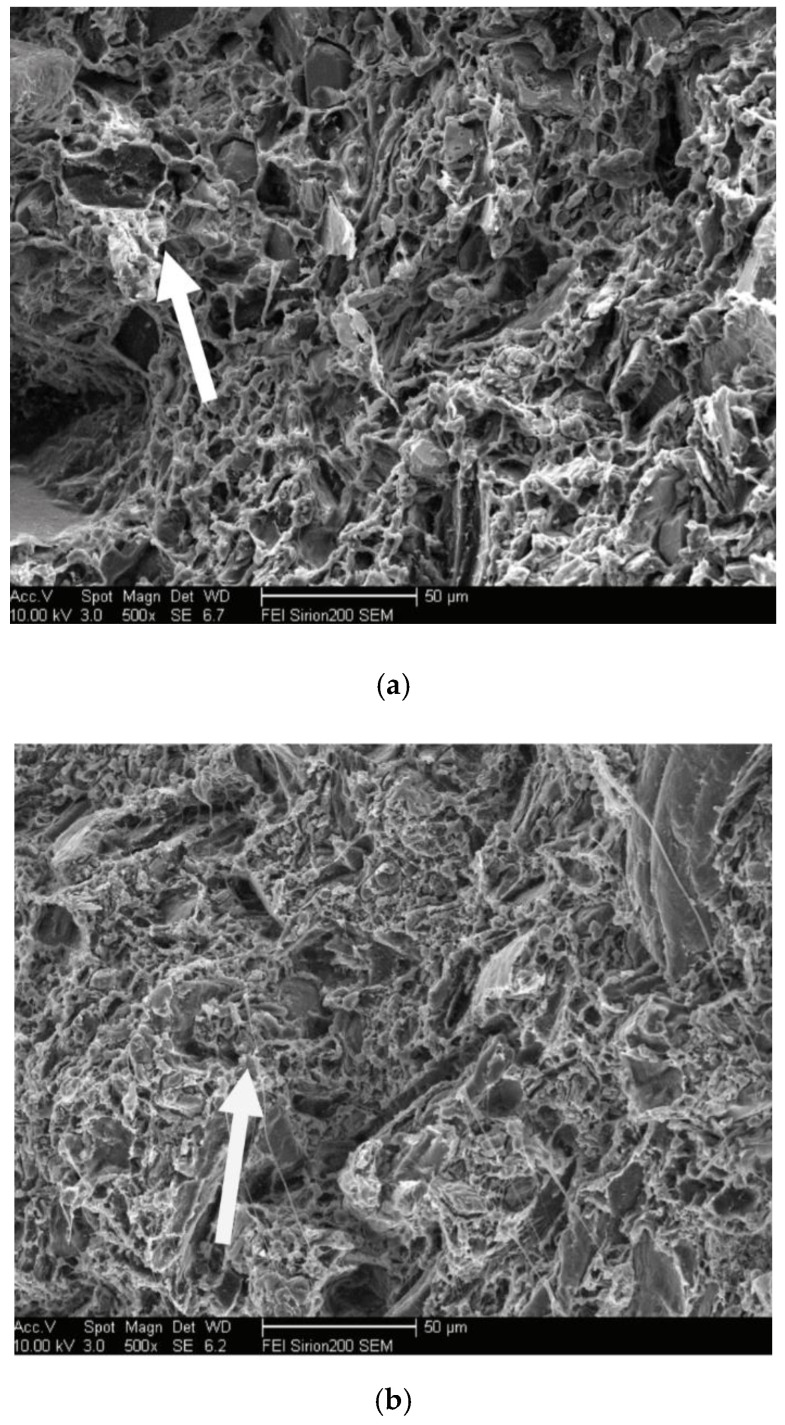
The SEM photos of WPC (adapted with permission from Cai et al. [37]): (**a**) without calcium carbonate (×500) and (**b**) added 10% calcium carbonate (×500).

Nano SiO_2_, TiO_2_, nano carbon fiber tube, and nano CaCO_3_ are the most widely used materials for WPC modification. The effect of calcium carbonate content on the preparation and mechanical properties of HDPE-based WPC was investigated by Cai et al. [37]. The results showed that the WPC had the best bending performance when the calcium carbonate content was 10%. The SEM photos of WPC impact fracture under 0% and 10% additive amounts of calcium carbonate are shown in Figure 5. It can be found that calcium carbonate can fill WPC well and make the internal structure more compact.

**Figure 6:**
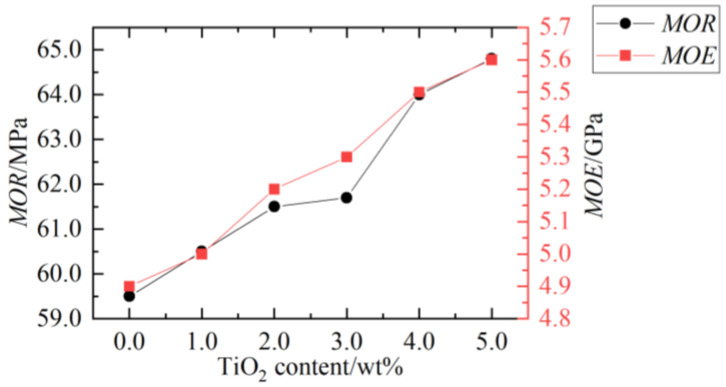
TiO_2_ content and flexural properties [64].

**Figure 7:**
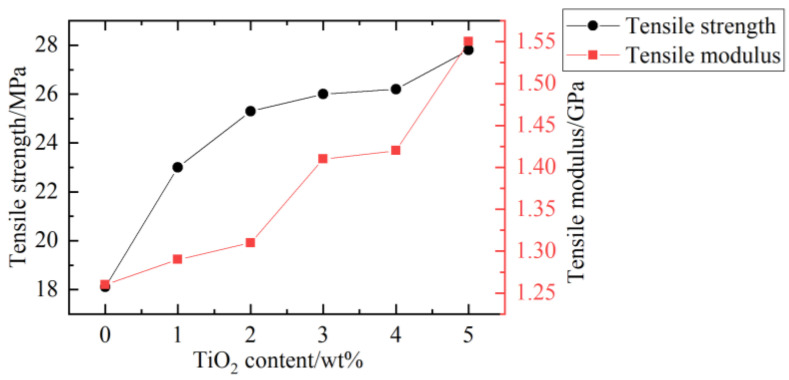
TiO_2_ content and tensile properties [64].

Kaymakci [64] studied the influence of loaded TiO_2_ on the mechanical properties of wood–plastic nanocomposites. Pine powder was compounded with maleic anhydride polypropylene, nano TiO_2_, and PP in a twin-screw rotary extruder. The flexural properties of WPC nanocomposites increased with the increase of titanium dioxide content as shown in Figure 6 and Figure 7. 

**Figure 8:**
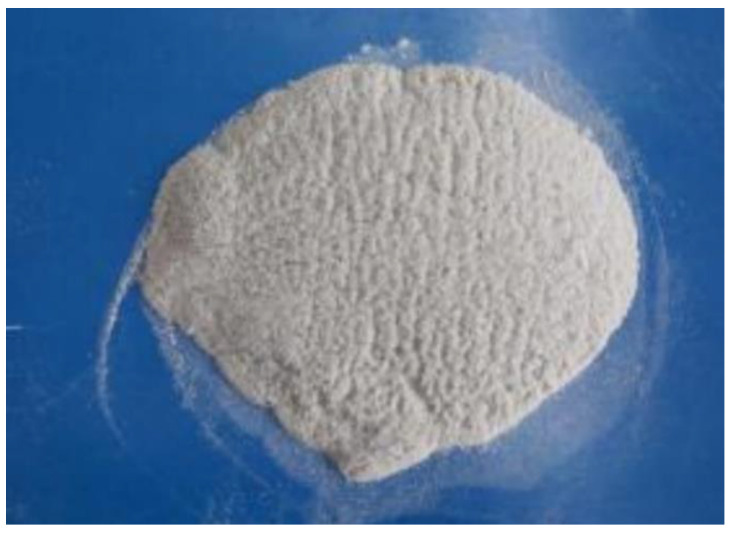
Optical images of the composite powder of wood flour, polyether sulfone (PES), and various amounts of 0.1% CNTs [84].

**Figure 9:**
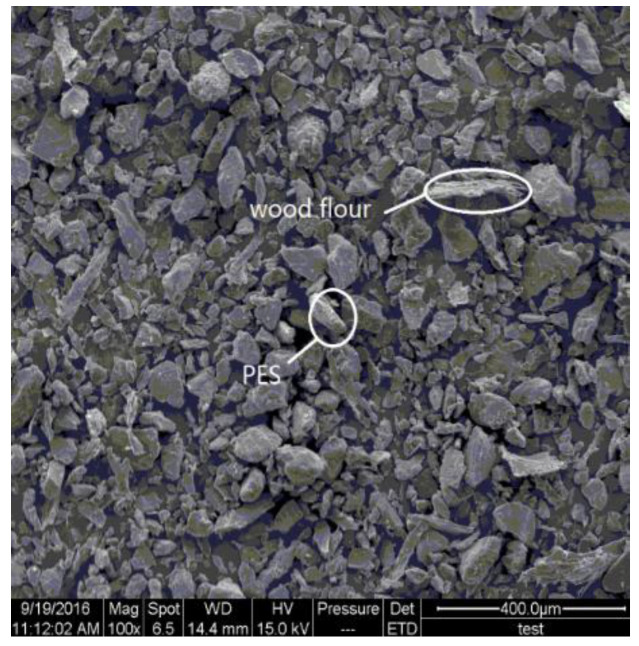
SEM image of the composite powder of wood flour, PES, and 0.1% CNTs [84].

**Figure 10:**
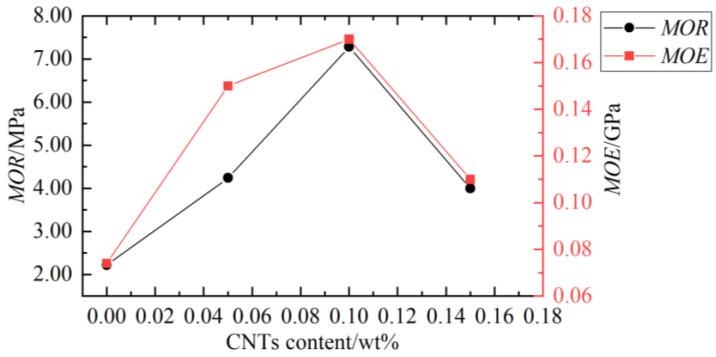
CNTs content and flexural properties [84].

Ghalehno et al. [83] used HDPE and pine fiber to prepare WPCs. The effects of adding TiO_2_ nanoparticles with different mass fractions on some properties of the composites were investigated. The results showed that the bending strength of WPC is improved by using nano TiO_2_ as reinforcement. In order to improve the mechanical properties of WPCs, Zhang et al. [84] prepared a new WPC containing carbon nanotubes (CNTs), wood fiber (14 wt.%), and polyethersulfone (86 wt.%). Images of the composite powder are shown in Figure 8 and Figure 9, while the flexural properties with different CNTs content are shown in Figure 10. The experimental results showed that adding a small amount of CNTs could significantly improve the mechanical properties of wood/PES composites. 

Through a comparative analysis, it can be found that the optimal amounts of nanomaterials to be added for best performance depend on the types of materials and wood fiber contents. Modification treatment of nanomaterials before addition can enhance the modification effect and obtain high-performance materials.

### 5.3. Modification by Adding Minerals

Adding other minerals, such as high-density soil, can improve the flexural properties of WPC. Zhang et al. [85] studied the influence of adding flake graphite on the mechanical properties of WPC and found that WPCs with FG filler had a large bending modulus but a low bending strength due to uneven stress distribution. In general, the mechanical properties of WPCs decreased slightly with the increase of FG content. Zhang et al. [86] studied the mechanical properties of WPC reinforced with HDPE hybrid reinforced with mineral filler and wood powder, and the results showed that the addition of mineral filler can improve the bending properties of WPC, especially the improvement of modulus. Li et al. [87] reinforced HDPE WPC with clay to study the influence of clay dosage on the properties of the prepared WPC composite. The results showed that both the bending strength and modulus of the composite increases as the clay content is increased up to 5% but excessive clay content has a negative impact on mechanical strength. In order to enhance WPC, Li et al. [88] embedded organic vermiculite (O-VMT) and triphenylbenzophenous chloride (BTPPC) into the vermiculite (VMT) as the reinforced filler to prepare WPC. The mechanical properties of the composites were studied. The results showed that WPC/O-VMT composites exhibit high bending properties.

Therefore, the types of added minerals and treatment methods, the determination of the optimum amount of added minerals, and the collocation of minerals with raw materials and the environment need to be further researched and developed.

### 5.4. Modification by Adding Non-Metallic Microfibers

The bending property of WPC can be improved by adding bamboo charcoal and plastic microfiber [89]. Zhang et al. [90] found that glass-fiber-reinforced WPC has a good interfacial bonding performance and that adding continuous fiber with low-volume fraction into WPCs could potentially make it into a load-bearing engineering material. Heriyanto et al. [91] used waste PP containers and sawdust to produce WPC and used mixed waste glass as a secondary filler, and they found that glass-reinforced WPC had a high hardness (MOE); adding glass powder every 5 wt.% could increase it to about 0.5 GPa. Li et al. [92] took bamboo charcoal (BC) as the reinforced filler of WPC and pretreated the bamboo–WPC. The influence of BC and water treatment on bending properties was studied. The results show that BC has a strong interface interaction in WPC, and BC–WPC has higher bending properties than WPC. Mosavi-mirkolaei et al. [93] studied the influence of recycled plastic microfiber blending on the physical and mechanical properties of WPC. The mixture was extruded and stretched to obtain microfiber morphology. The addition of PET microfiber improved the mechanical properties of the sample. The bending modulus of PET composites was reported to be 9% higher than that of composites without PET. Zhang et al. [94] used flax fiber (FF) to reinforce wood flour/high-density polyethylene composite (WF/PE). It was found that the mechanical properties of the composites can be significantly improved by adding a small amount of FF. The flexural strength and modulus were increased by 14.6 and 51.4% (FF content was 9 wt.%), respectively. 

As a building component, WPC can be reinforced with FRP to improve its flexural performance [95]. Wang et al. [96] studied the effects of bakelite extract on mechanical properties and anti-fungal corrosion of bakelite based WPMC. The results showed that the mechanical properties of xylan prepared with extracted RWF were higher than those of unextracted xylan. Kumar et al. [97] added PP with mango/sheesham/mahogany/babool dust in different proportions to improve mechanical properties. When wood chips (10%, 15%, and 20% by weight) were mixed with PP particles, it was found that the WPC composed of babool and sheesham dust had good mechanical properties. FRP wrapped around the outside of the biomass material component can also play a role in strengthening [98]. 

Nonmetal can also be used as a part of the composite components to enhance the flexural properties of WPC. After processing, biomass materials can be constructed into offices and other buildings [99]. Zong et al. [100] prepared a new WPLC by using high-strength light laminated single sheet (LVL) as the core material and water-resistant wood–polyvinyl chloride composite (WPVC) as the shell layer. The wood fiber content was 60 wt.%, and the comparison of WPLC60 and WPVC showed that the specific bending modulus of WPLC60 was 284% of WPVC60. WPC produced using co-extrusion technique has a special shell structure. Its shell layer is completely coated with the core layer, and additives with different functions are added to the shell layer to improve the mechanical properties of the WPC [101]. Siqueira D.D. et al. found that the exudation of macaiba oil to the surface reduces the interaction with water and thus increases the contact angle, while the time and polarity effects alleviate the entry of water from the surface into the composite core and improve the interaction between PCL matrix and macaiba [102].

To enhance the flexural performance of WPC, surface-pretreatment research mainly concentrates on the anhydride-grafted monomers, namely the impact of the type of compatibilizer content on the enhancement effect; and there is an optimum quantity for the added material. The influence of the reaction of various compatibilizers with the plastic matrix and lignocellulosic fiber on the strengthening effect, so as to select the best combination of fiber, plastic, and graft monomer, needs further and more comprehensive research. For the enhancement methods of modifying agents and substances with other effects, further research should be conducted considering different types and processing methods of charcoal, bamboo charcoal, high-density soil, and minerals. By improving the raw material and ratio of WPC, selecting the best particle size, and adding a reasonable type and ratio of compatibilizers, WPCs can have a more extensive and meaningful applications in the field of bending components. Although composite materials can combine the strength of wood with the toughness of plastic, there is still a certain gap compared with the advantages of pure wood and plastic. In addition, how the processing cost of composite materials can meet the needs of engineering needs further research. Table 2 shows the summary of modification methods and mechanical properties, Table 3 shows the summary of raw materials of WPC in this paper and provides references for future research.

## 6. Conclusions

This paper reviewed and analyzed all influencing factors and modification methods for improving the bending properties of WPC. The bending properties of WPCs made from different materials were summarized, and the effects of different modification methods were compared and analyzed as part of the current study. The main conclusions are as follows:

(1) The internal factors affecting the mechanical properties of WPC primarily include the types and contents of wood, plastic matrix, and compatibilizers. It was observed that the WPC with wood as poplar and Chinese fir and plastic matrix as PP, PS, and HDPE offer good bending property. 

(2) The flexural property of WPC can be improved by both physical and chemical pretreatment of raw materials. Further research is required to find the optimum amount of various grafted copolymers. The processing technology and the selection of a suitable technology to add grafted monomers are still not fully explored. The interaction between grafted monomers and LFC and plastic matrix should also be investigated through experimental research.

(3) Addition of modifiers has shown obvious effects on WPCs mechanical properties. Modifiers mainly include compatibilizers containing anhydride and carboxyl group, and coupling agents containing silane and titanic acid, lubricants, surfactants, etc. The type and content of modifier should be determined according to the nature of the material and the conditions of use.

(4) Nano silicon dioxide, nano titanium dioxide, nano carbon fiber tube, nano calcium acid, and other nano materials can fill the gap between wood fibers to enhance the compactness of WPC and, hence, enhance its mechanical properties. Research on exploring new nanomaterials for possible application in WPC production would be a timely research to combat plastic waste. At present, most of the research on WPC focuses on the processing-method-and-raw-material ratio. Further research is needed on the compressive and shear properties of WPC to promote its wider use in engineering applications.

## Figures and Tables

**Table 1:** Summary of the relationship between raw material types used in WPC and the relevant flexural properties.

References	Wood	Plastic Matrix	Wood Fiber Content (wt.%)	Bending Strength (MPa)	Bending Modulus (GPa)
[35]	Pinus massoniana	HDPE	60	43.3	3.2
[35]	Chinese fir	HDPE	60	63.9	4.0
[36]	Mechanical pulp of bagasse	HDPE	50	65.9	-
[50]	Scottish loose	PP	50	24.8	3.784
[51]	Populus amurensis	PP	38	40.5	1.891
[42]	Populus amurensis	HDPE	59.7	59.27	2.73
[44]	Pinus densifora	PP	25	-	3.6
[45]	Sengon sawdust	HDPE	-	40.39	3.353
[46]	Thermomechanical wood fibers	PP	50	76.4	5.2
[49]	Spruce and pine	PS	75	35	5.1
[59]	Commercial wood flour	PP	25	73.7	2.59
[3]	Rubberwood flour	LDPE	40	6.39	1.0
		HDPE	60	18.79	0.83
		PP	60	37.72	2.17
		PVC	60	15.6	0.95
		PS	50	30.03	3.51
[60]	Poplar wood	rPP/ PE	60	52.4	6.06
		rPP/PE/PS		52.9	5.49
[4]	Saguvani	Phenol formaldehyde (PF)			
[61]	Wood flour	PLA	18.5	95.3	-
[62]	Pine powder	Polyethersulfone resin (PES)	14.3	16.8	-
[63]	Poplar	HDPE	60	27.47	2.4
[64]	Pinus sylvestris	PP	50	64.8	5.6
[65]	Rice husk	PLA	30	11.27	
	Bamboo powder			14.17	
	Poplar powder			16.26	
[66]	Chili-stems waste particles	PP	21.25	22.9	0.024
			42.5	16.2	0.0255
[67]	Bark flour	HDPE	50		
	Beech-wood flour		50		

**Table 2:** Summary of modification methods and mechanical properties.

References	Wood	Plastic Matrix	The Amount of Wood Fiber (wt.%)	Modification Method	The Increase in Bending Strength (%)	The Increase in Bending Modulus (%)
[12]	Wood flour (80 mesh)	HDPE	50	Hydrophobic modification of Methyl methacrylate (MMA)	17.3	24.4
				Butyl methacrylate (BMA)	26.3	24.4
				Styrene (St)	27.5	26.0
[17]	Coir	PP	5–15	The coir is treated with 17.5% alkali for 1 h	10	-
[18]	Wood flour (80 mesh)	PP	38.48	The addition of nanosilica (1.52 wt.%)	-	-
[19]	poplar wood (60–80 m)	HDPE	50	high-temperature hot air (HTHA) treatment and silane coupling agent	-	-
[21]	Poplar flour (60 m)	HDPE	30	Addition of optimized glass fiber (GF, 15%)	6.8	-
[37]	Eucalyptus wood	recycled polyethylene (rPE)	50	Addition of ternary-monomer graft copolymers		
[62]	Pine powder	Polyethersulfone resin (PES)	14.3	Micron-scale aluminum powder is added (0.1 wt.%)	130.36	-
[63]	Poplar (80–100 m)	HDPE	60	Multi-walled carbon nanotubes were added (0.5 wt.%)	5.8	13.7
[64]	Pinus sylvestris	PP	50	Nano-TiO_2_ was added (5 wt.%)	8.9	14.3
[65]	bamboo powder	PLA	30	Silane coupling agent was added	-	-
[75]	Populus adenopoda, 40–80 mesh	HDPE	80	Ultra-highly filled wood fiber/PE composites (UH-WPCs) was fabricated by using maleic anhydride grafted polyethylene (MAPE) as compatibilizer	189	
[76]	poplar	HDPE	50	Impulse-cyclone drying (ICD) and 3% silane was added	10.22	
[77]	Alkali lignin and Eucalyptus powder	HDPE	Alkali lignin:15 Eucalyptus powder:45	Eucalyptus powder was replaced with coffee shells	15.30	-
[83]	Pinus sylvestris	HDPE	50	Nano-TiO_2_ was added (3 wt.%)	21.88	-
[84]	pine powder	(PES)	14	Carbon nanotubes was added (0.1 wt.%)	227.9	128.7
[94]	Poplar (40–80 m)	HDPE	51	Flax fiber (FF) was added (9 wt.%)	14.6	51.4

**Table 3:** Summary of wood–plastic composite composition.

Wood Fiber	Plastics Substrate	Modifying Agent
Rubberwood [3] Saguvani [4] Coconut chaff [17] Beech wood [31] Radiata pine [33] Wheat straw [33] Moso bamboo [33] Poplar fiber [34] (endothelial powder, skin powder) Bamboo fiber [34] Masson pine wood meal [35] Chinese fir wood meal [35] Pinus massoniana [35] Bagasse [36] Eucalyptus globulus particles [37] Spruce [38] Rice husk [41] Populus amurensis [42] Pinus densifora [44] Sawdust [45] Thermomechanical wood fibers [46] Foam core particleboard [49] Decayed wood powder [50] Scottish loose [50] Pepper leaf [65] Chili-stems waste particles [65]	Low density polyethylene (LDPE) [3] High density polyethylene (HDPE) [3] ABS [3] PS [3] PP [3] PVC [3] Recycled polypropylene (rPP) [40] Polylactic acid (PLA) [47] Polymethyl methacrylate (PMMA) [69] Poly (ether sulfone) [81]	Nano silica [18] Glass fiber [21] Maleic anhydride [37] Calcium carbonate [37] Polyethylene glycol (peg) [47] Acrylic acid [48] Aluminum powder [61] Carbon nanotube [62] Toluene-2, 4-diisocyanate (TDI) [69] Trimethoxysilane (MPTMS) [69] Coffee shell powder [74] Nano clay [78] Sepiolite clay nanofibers [79] Flake graphite [82] Organic vermiculite [85] Bamboo charcoal [86] Recycled plastic microfibers Flax fiber [91] Macaiba [99]
